# Spatial epidemiology of dengue and chikungunya in Karnataka using GIS-based analysis

**DOI:** 10.1080/16549716.2025.2543198

**Published:** 2025-08-21

**Authors:** Prathiksha Prakash Nayak, Jagadeesha Pai B, Sreejith Govindan, Vaishnavi Sanagapalli

**Affiliations:** a Department of Civil Engineering, Manipal Institute of Technology, Manipal Academy of Higher Education, Manipal, India; b Division of Microbiology, Department of Basic Medical Sciences, Manipal Academy of Higher Education, Manipal, India; c Department of Applied Statistics and Data Science, Prasanna School of Public Health, Manipal Academy of Higher Education, Manipal, India

**Keywords:** Dengue, Chikungunya, spatial autocorrelation, Geographic Information, Spatial Epidemiology System (GIS)

## Abstract

**Background:**

Dengue and chikungunya are major vector-borne diseases with significant public health concerns. Understanding their spatial distribution is crucial for effective disease control and prevention strategies.

**Objective:**

To analyse the spatial patterns and trends of dengue and chikungunya in Karnataka from 2021 to 2024 using GIS-based methods to identify high-risk districts.

**Methods:**

Spatial autocorrelation analysis using Moran’s I and cluster analysis was performed to examine the spatial distribution of dengue and chikungunya incidence rates using GIS-based mapping.

**Results:**

A total of 38,229 dengue cases and 8,094 chikungunya cases were recorded during this period, with the highest incidence rates varying across districts. The highest dengue IR in 2024 was recorded in Chikkamagaluru (0.58/1000), Mandya (0.47/1,000), followed by Udupi, while Vijayapura reported the highest chikungunya IR in 2022 (0.17/1,000). Moran’s I result revealed moderate positive autocorrelation for dengue in 2021 and 2023, indicating significant clustering of cases. By comparison, chikungunya exhibited negative spatial autocorrelation in 2023 and a shift to positive in 2024, reflecting a transition from dispersed to more clustered patterns.

**Conclusion:**

GIS-based surveillance enhances early outbreak detection, resource allocation, and targeted vector control. Spatial autocorrelation techniques support predictive modelling for other infectious diseases. Strengthening interdisciplinary collaboration, community engagement, and spatial analytics in public health policies supports disease mitigation and urban planning. Globally, integrating spatial epidemiology into surveillance systems can improve outbreak prediction, optimise resources, and guide data-driven policy. These findings highlight the need for GIS-based frameworks and international cooperation to address emerging disease threats effectively.

## Paper Context

**Main findings:** Dengue incidence peaked in 2024 with pronounced spatial clustering in high-risk districts like Chikkamagaluru, Mandya, and Udupi, while chikungunya showed weaker, shifting patterns, highlighting divergent transmission dynamics across Karnataka.

**Added knowledge:** Integrating spatial epidemiology with GIS-based cluster analysis enhances understanding of vector-borne disease transmission and improves outbreak prediction.

**Global health impact for policy and action:** GIS-integrated public health strategies can strengthen real-time surveillance, targeted interventions, and global collaboration for effective disease control.

## Background

Dengue is caused by the dengue virus (DENV), which causes a spectrum of illnesses ranging from undifferentiated fever to asymptomatic or mild febrile illness, dengue fever (DF), dengue haemorrhagic fever (DHF), and dengue shock syndrome (DSS) [1]. DENV is considered the most critical arbovirus; the most severe cases are caused by four distinct serotypes, i.e. DENV-1, DENV-2, DENV-3, and DENV-4, now circulating in Asia, Africa, and the Americas [2]. The endemicity levels of DF reflect the distribution of its primary urban mosquito vectors, *Aedes aegypti* and *Aedes albopictus*, to new geographical areas, warm and humid climates, increased population density, water storage pattern in houses, storage of junk in open spaces, including tyres, coconut shells, etc. that trap rainwater and introduction of new serotype of the virus [3,4]. The prevalence of dengue in India is reported to be 289,235 cases, with 485 deaths in 2023, highlighting the country’s substantial contribution to the global dengue burden and the ongoing challenges in disease management and prevention [5]. Dengue transmission is influenced by complex interactions among the host, vector, and virus, all affected by climatic factors [6].

The Sixth Assessment Report (AR6) by the Intergovernmental Panel on Climate Change (IPCC) provides detailed insights into climate change’s global and regional impacts, including Asia, the Indian Ocean, India, and its subregions like Karnataka [7,8]. Asia faces significant risks, including flooding, heat waves, and water scarcity. Urban areas in South and Southeast Asia are particularly vulnerable. The Indian Ocean region is experiencing accelerated sea level rise, warming, and marine ecosystem degradation, threatening coastal communities. India’s vulnerability is linked to its high population density and dependence on climate-sensitive sectors like agriculture and water resources. The Southwest monsoon is becoming erratic, affecting food and water security. Coastal regions are increasingly exposed to cyclones and sea-level rise. India’s position emphasises climate justice, urging developed countries to fulfil financial and technological support commitments. The Western Ghats, covering parts of Karnataka, are recognised as a biodiversity hotspot but face threats from climate change, including shifting rainfall patterns and rising temperatures. Karnataka’s monsoon patterns are increasingly unpredictable and inconsistent, with evidence of intensified rainfall and shifting seasonal onset, which directly influence the breeding habitats of *Aedes* mosquitoes. Such climate-induced changes contribute to increasing the incidence and spatial spread of dengue and chikungunya cases in the region [4,9]. The South Asian climate is heavily influenced by the Asian monsoon, with India receiving 75% of its rainfall during the southwest monsoon season from June to September. In tropical regions like India, dengue exhibits strong seasonality, yet limited research has assessed the impact of climatic factors on dengue transmission [10]. Countries like Singapore and Thailand have successfully managed outbreaks early using tools like GIS for hotspot mapping and field data collection. Strategies include mosquito surveillance, redesigning habitats, and adopting innovative technologies like heated roof gutters and behaviour tracking [11]. Temperature also plays a role, as *Aedes aegypti* mosquitoes thrive between 25°C and 30°C, while extreme temperatures below 15°C or above 35°C reduce their survival [1].

Similarly, chikungunya virus (CHIKV) poses a significant public health threat, particularly in non-endemic regions with competent *Aedes* vectors. Despite its increasing prevalence and the re-emergence of chikungunya, limited studies have explored its economic impact, highlighting a critical gap in understanding the broader consequences of the disease [12–15]. The similarity between the symptoms of the two important arboviral diseases (dengue and chikungunya) and the absence of laboratory confirmation leads to misclassification bias [16]. Over the past decade, CHIKV has emerged and re-emerged in several regions, including Kenya (2004), Comoros (2004, 2007), Seychelles (2004, 2006), Mauritius (2005), La Réunion (2005, 2007), and India (2005) [17]. CHIKV is believed to have transmission patterns like DENV since the same mosquito vectors spread both. These patterns often lead to clusters of cases in and around the households of infected individuals due to the anthropophilic nature of *Aedes*, which typically disperses over short distances and remains near homes. Beyond this range, human movement plays a key role in spreading DENV, and similarly, high population density, especially in urban areas, has been linked to the clustering of chikungunya cases [18]. The global surveillance network reports that common travel destinations associated with chikungunya infections include the Indian Ocean Islands, Brazil, Thailand, India, Malaysia, Maldives, and Myanmar. Between epidemics, the CHIKV persists in mosquito and animal reservoirs, such as nonhuman primates. Transmission occurs year-round in tropical and subtropical regions, with increased activity during the rainy season. The disease burden is influenced more by contextual factors, such as dwelling type, altitude, household size, and area of residence, than by individual characteristics like gender, age, occupation, or preventive behaviours [15,19,20].

The rapid advancement of GIS has significantly enhanced the role of spatial data analysis in understanding and managing infectious disease epidemiology [21]. Given the geographical diversity and varied disease burden across Karnataka’s districts, GIS can be highly effective for mapping the spatial distribution of dengue and chikungunya. Mapping the data can help identify high-risk areas, understand how the disease correlates with environmental factors like land use, population density, and climatic conditions, and improve resource allocation for targeted interventions in the most affected regions [22,23]. GIS-based mapping and spatial statistical analysis have become crucial tools in monitoring and managing vector-borne diseases like dengue and chikungunya. Over time, geoprocessing and digital mapping techniques have been used to analyse public health issues, visualise epidemiological data, and predict disease risks. Spatial statistics, such as cluster analysis, hotspot analysis, and spatial autocorrelation, help understand the spatial distribution and environmental factors associated with disease outbreaks. Spatial and temporal cluster analyses can guide highly effective, locally targeted interventions for disease control with significant spatial variation [24–27].

Although few studies in India have explored the spatial distribution of dengue and chikungunya using GIS tools, most have been confined to specific cities or taluk-level regions, often relying on outdated or single-year datasets and focusing on only one disease [28,29]. In Karnataka, there is a notable lack of comprehensive district-level analyses that assess the spatial dynamics of both dengue and chikungunya simultaneously using recent post-2020 data. Existing governmental surveillance data often lacks geocoordinate precision, limiting the ability to perform fine-scale spatial analysis. As a result, spatial clustering patterns and hotspots remain underexplored at actionable scales. This study addresses these critical gaps by integrating publicly available health data with GIS-based spatial statistical methods, including Moran’s I and Local Indicators of Spatial Association (LISA), to examine clustering patterns and potential environmental influences across all districts in Karnataka from 2021 to 2024.

## Methods

### Study area

**Figure 1.**
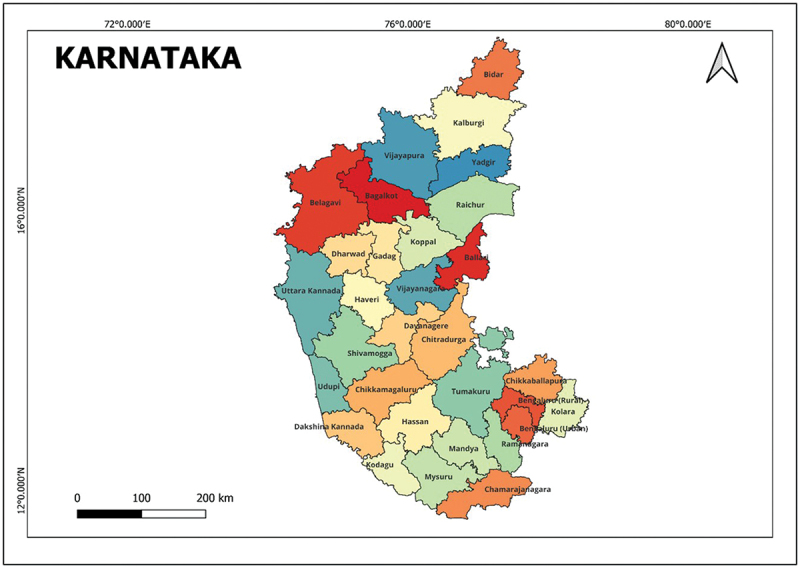
Study area- Karnataka state.

Karnataka state is located in the southwestern region of India, bordered by the Arabian Sea to the west and surrounded by Goa, Maharashtra, Telangana, Andhra Pradesh, Tamil Nadu, and Kerala. It is the sixth-largest state in India by area, and as of the last census report (conducted in 2011), it has a population of over 61 million people. The state has 31 districts (Figure 1).

### Data collection

Epidemiological data: Data on dengue and chikungunya cases were sourced from the National Vector Borne Disease Control Programme (NVBDCP) reports, publicly available through the Ministry of Health and Family Welfare’s official website [30]. Data for dengue and chikungunya were collected from January 2021 to December 2024. All dengue and chikungunya cases were confirmed using IgM MAC-ELISA (Immunoglobulin M Antibody Capture Enzyme-linked Immunosorbent Assay) and NS1 (Non-Structural Protein 1) antigen detection tests, as per NVBDCP guidelines.

**Incidence rate (IR)**: Population estimates were used to compute the IR per 1000 individuals.

IR was calculated for each district as:

$$\mathrm{Incidence}\;\mathrm{Rate}\;\left(\mathrm{IR}\right)=\frac{\mathrm{Confirmed}\;\mathrm{cases}}{\mathrm{Total}\;\mathrm{population}}X1000$$

Incidence rates were calculated using adjusted population estimates for each district, derived by applying growth correction factors based on the decadal growth rates from the 2001 and 2011 censuses, as reported in the official district-wise statistics from the Government of Karnataka [31].

**Mapping disease endemicity**: Spatial autocorrelation techniques are essential in understanding the geographic distribution of disease IR. In this study, Moran’s I and Local Indicators of Spatial Association (LISA) examine spatial clustering patterns of dengue and chikungunya IR from 2021 to 2024 across Karnataka.

**Spatial weights matrix**: A spatial weights matrix was constructed to conduct spatial autocorrelation analysis. This matrix defines the spatial relationships among districts based on either contiguity (Queen or Rook adjacency) or distance-based methods.

## Spatial autocorrelation analysis: local Moran’s I

Moran’s I is a global spatial autocorrelation measure that quantifies the degree of similarity among spatial units based on their attribute values and geographic proximity. It is computed as follows:

$$\mathrm{Ii}=\frac{\left(Xi-\overset{ˉ}{X}\right)}{S^{2}}\underset{j}{\sum }\mathrm{wij}(\mathrm{Xj}-\overset{ˉ}{X})$$

where:

*Ii* = Local Moran’s I statistic for location *i*

*Xi* = Value of the variable at location *i*

$\overset{ˉ}{X}$ = Mean of the variable across all locations

*S*^*2*^ = Variance of the variable

*wij* = Spatial weight between location *i* and *j*

*Xj* = Value of the variable at neighbouring location *j*

For this study, Moran’s I for dengue and chikungunya IR from 2021 to 2024 were calculated. The statistical significance of Moran’s I was assessed using a permutation test with 999 randomisations.

## LISA analysis

While Moran’s I provides a global measure of spatial clustering, it does not reveal localised patterns. To overcome this, LISA was employed to detect spatial clusters (hotspots and cold spots) and spatial outliers. LISA statistics are derived using the following formula:

$$\mathrm{Ii}=\mathrm{Zi}\underset{j}{\sum }\mathrm{wijZj}$$

where:

*Ii* = Local Moran’s I for location *i*

*Zi*  = Standardized value at location *i*

*Zj* = Standardized value at neighbouring location *j*

*wij* = Spatial weight between location iii and *j*

$\underset{j}{\sum }\mathrm{wijZj}$ = Spatially weighted sum of neighbouring values

**Software tools**: Spatial analysis was conducted at the district level (31 administrative units) using shapefiles of Karnataka obtained from Karnataka Geographic Information System (K-GIS), developed by the Karnataka State Remote Sensing Applications Centre (KSRSAC) [32]. All spatial data layers were projected in the WGS 1984 geographic coordinate system (EPSG:4326) to ensure consistency in analysis and visualisation across platforms. Mapping and data visualisation were performed in QGIS (version 3.36.3), while spatial statistical analyses were conducted in GeoDa (version 1.22). A spatial weights matrix was constructed using the first-order queen contiguity method, where districts sharing a boundary or vertex were considered neighbours. To ensure full connectivity among all spatial units, a threshold distance of 250 km was also considered during spatial weight construction.

## Results

**Figure 2.**
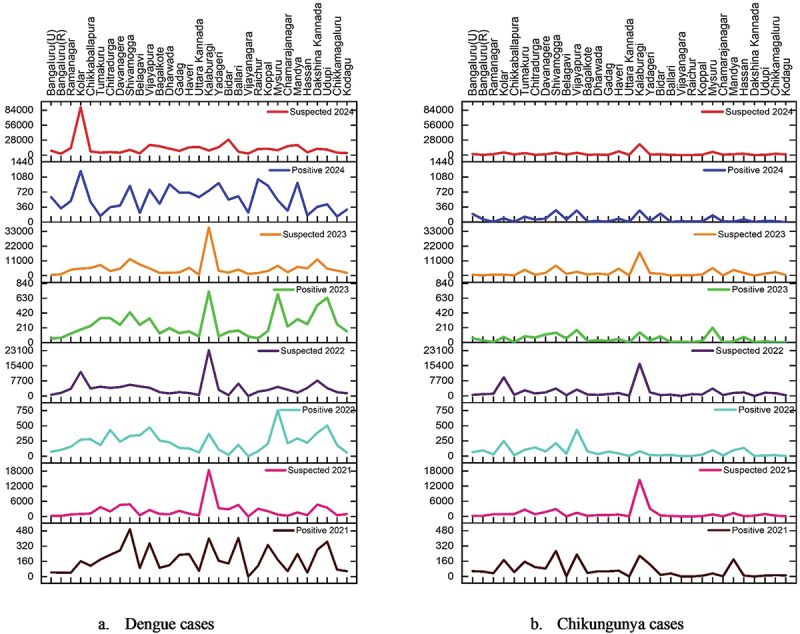
Suspected and positive cases of dengue (a) and chikungunya (b) during 2021–2024.

The epidemiological data of Karnataka reveals a significant burden of dengue cases from 2021 to 2024. A total of 38,229 dengue cases were reported across 31 districts. The year-wise distribution shows 5619 positive cases in 2021, 7307 cases in 2022, 7930 cases in 2023, and a sharp increase to 17,373 cases in 2024; chikungunya cases account for 8,094 positive cases from 2021 to 2024, as shown in Figure 2.

## Dengue IR in the state of Karnataka

**Figure 3.**
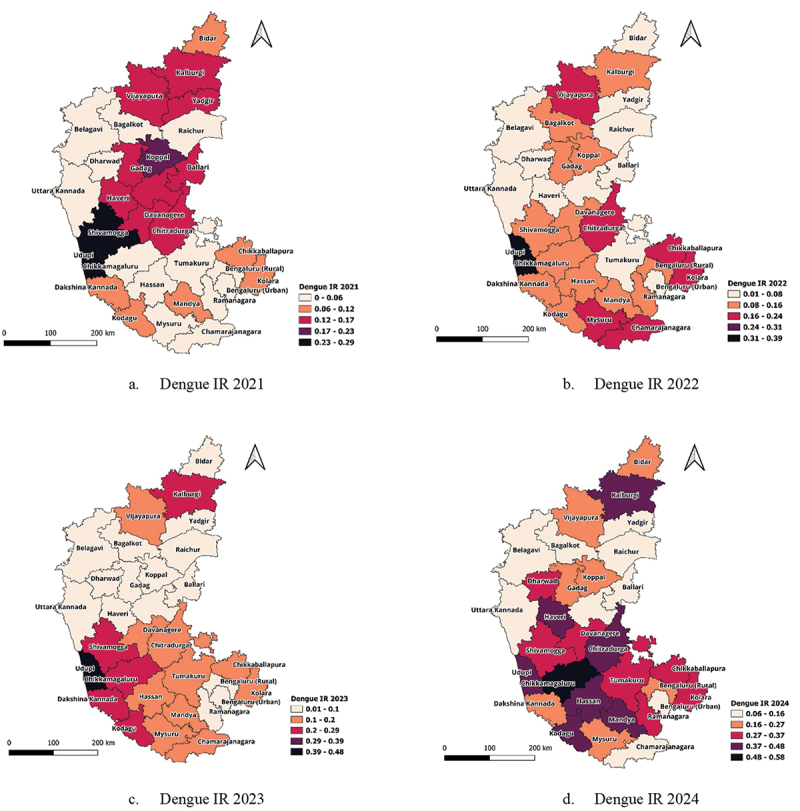
Dengue IR (a-d) for 2021–2024.

The dengue IR across the 31 districts of Karnataka exhibited significant spatial and temporal variations between 2021 and 2024. The highest IR in 2024 was recorded in Chikkamagaluru (0.58 per 1,000 population), followed by Mandya (0.47 per 1,000) and Udupi (0.45 per 1,000), indicating a substantial increase in disease burden in these districts, as shown in Figure 3. In contrast, Bengaluru Urban consistently reported the lowest IR values, ranging from 0 in 2021 to 0.06 in 2024, suggesting a relatively lower dengue transmission in the state’s capital.

Several districts experienced a sharp increase in IR over time, with some showing nearly a threefold rise. Hassan recorded an IR of 0.02 in 2021, which surged to 0.41 in 2024, while Chikkamagaluru exhibited a rise from 0.06 in 2021 to 0.58 in 2024. Similarly, Ramanagar’s IR increased from 0.03 in 2021 to 0.32 in 2024, signifying an expanding transmission pattern. Other districts, such as Udupi and Chikkamagaluru, also showed a consistent rise in incidence, reaching 0.45 and 0.58 per 1,000, respectively, by 2024. A particularly notable trend was observed in Udupi, which reported the highest dengue IR in Karnataka for three consecutive years. In 2021, Udupi’s IR was 0.29, rising sharply to 0.39 in 2022, and 0.48 in 2023 making it the high-risk spot in the state that year. However, in 2024, the IR declined slightly to 0.45, indicating a potential stabilisation or impact of intervention measures. Udupi’s trend highlights a concerning yet fluctuating pattern of dengue transmission, where the district consistently reported high incidence despite slight annual variations. These fluctuations may reflect changes in environmental conditions, vector control efficiency, or differences in dengue surveillance and reporting.

In contrast, certain districts displayed a fluctuating trend in dengue incidence. Bidar, Gadag and Mysuru saw variations in their IR values across the years, reflecting possible changes in vector control effectiveness, climatic influences, or healthcare response. While some districts, such as Raichur, Uttar Kannada and Belgavi, maintained relatively lower IRs throughout the study period, others, including Shivamogga, Tumakuru, Kolar, and Chikkaballapura, demonstrated a gradual increase in IR, suggesting a potential spread of dengue in these regions.

A higher dengue burden was evident in the southern districts when compared to northern Karnataka. Mandya, Shivamogga, Chikkamagaluru, Udupi and Mysuru consistently reported high IR values, significantly increasing in 2024. However, some northern districts such as Kalaburagi, Koppal, Vijayapura, Dharwad and Haveri also showed an upward trend in incidence, indicating that dengue is not confined to specific climatic zones but is progressively affecting a broader geographical region, as shown in Figure 3.

## Chikungunya IR in the state of Karnataka

**Figure 4.**
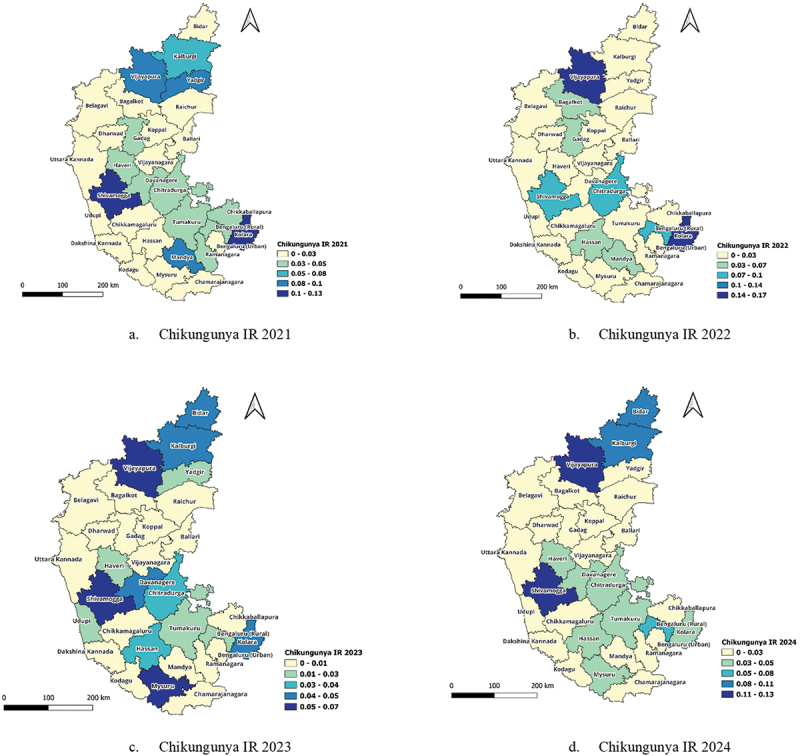
Chikungunya IR (a-d) for 2021–2024.

IR of chikungunya across the 31 districts of Karnataka from 2021 to 2024 shows a relatively stable pattern, with minor fluctuations over the years. Certain districts consistently reported higher IR values, while others showed declining trends or sporadic increases. Kolara, which had a high IR of 0.11 in 2021, declined to 0.05 by 2024, indicating a slight but consistent decrease in cases. Similarly, Mandya recorded an IR of 0.09 in 2021, which declined to zero by 2024, suggesting a significant reduction in chikungunya transmission., as shown in Figure 4.

Districts such as Shivamogga, Kolara and Vijayapura maintained relatively high IR values throughout the study period. Chitradurga recorded an IR of 0.05 in 2021, slightly decreasing to 0.03 by 2024, while Davanagere exhibited a similar trend, with a minor decline from 0.04 in 2021 to 0.03 in 2024. Vijayapura and Shivamogga also showed high IRs, remaining above 0.05 across the years, suggesting that these regions continue to experience a sustained chikungunya burden. Several districts experienced minimal fluctuations in IR, like Bengaluru urban and Raichur, and maintained nearly constant IR, with only a marginal decline of 0.01 per 1,000 annually. Other districts, such as, Gadag, Belagavi and Udupi, exhibited similar trends, with IRs remaining stable across the four years. Hassan, which had no reported cases in 2021, saw a sudden increase to 0.06 in 2022, maintaining this level through 2024. A similar trend was observed in Kodagu, where the IR remained zero until 2022 but rose to 0.02 in 2024, indicating the emergence of chikungunya cases in the region (Figure 4). Some districts demonstrated a steady decline in chikungunya IR, suggesting a gradual reduction in disease transmission. Districts such as Shivamogga, Chitradurga, and Davanagere showed slight decreases in IR over time, though the reduction was not as pronounced as in Mandya.

Overall, the spatial and temporal analysis of chikungunya incidence in Karnataka suggests that while some districts continue to experience persistent transmission, others have seen either a gradual decline or the emergence of new cases in previously unaffected areas. The stability in IR for most districts indicates that chikungunya remains endemic in Karnataka, with certain hotspots experiencing sustained transmission.

## Spatial autocorrelation of dengue incidence rates (2021–2024)

**Figure 5.**
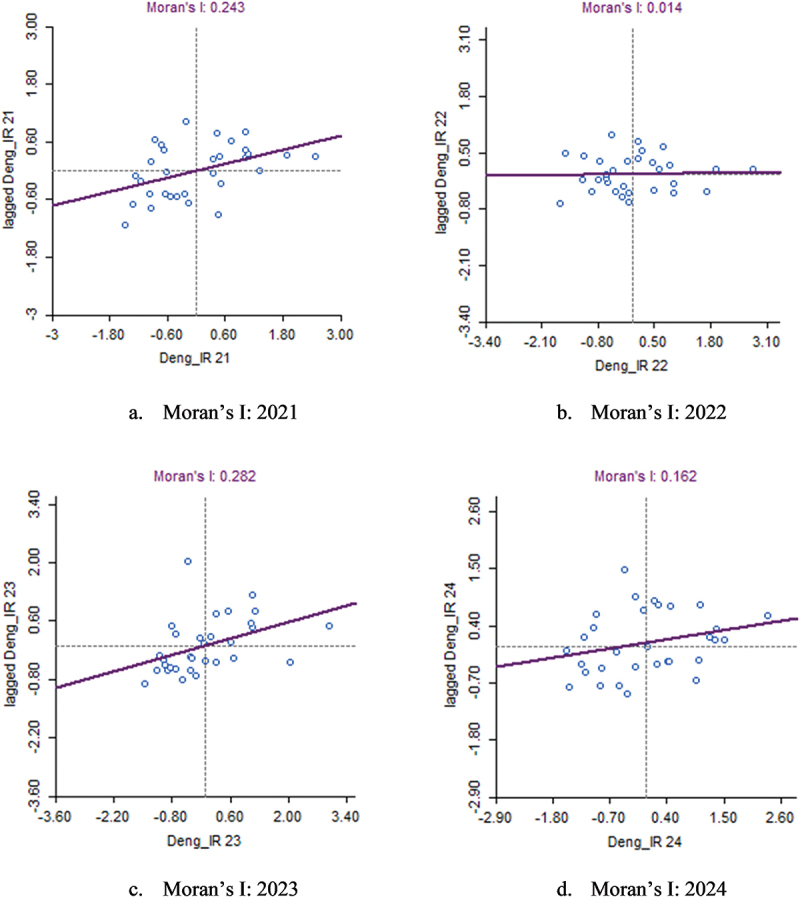
Moran’s I scatter plots (a-d) for dengue IR (2021–2024).

The Local Moran’s I statistic was used to detect significant spatial autocorrelation in dengue IR from 2021 to 2024. To assess this, Univariate Local Moran’s I was calculated and interpreted for each year as shown in Table 1, yielding values of 0.243 (2021), 0.014 (2022), 0.282 (2023), and 0.162 (2024), as shown in Figure 5. The results indicate moderate positive spatial autocorrelation in 2021 and 2023 (Table 2) meaning that districts with similar dengue IRs tended to be near each other, forming distinct clusters of high or low incidence. However, in 2022, Moran’s I value was 0.014, suggesting a nearly random spatial distribution of dengue cases with no strong clustering patterns, as depicted in Figure 5. In 2024, the Moran’s I value was positive (0.162), indicating that the observed clustering was not statistically significant, reflecting a weaker and less reliable spatial structure compared to 2021 and 2023. Moran’s I scatter plot visually represents these findings for each year (Figure 5). In 2021 and 2023, the scatter plots display a positive trend, confirming that districts with high dengue incidence are spatially clustered together, as are districts with low incidence. Conversely, in 2022, the scatter plot lacks a clear trend, and 2024 shows a mild, non-significant pattern, reinforcing the random distribution of dengue IRs across the state. These results suggest temporal shifts in spatial clustering, highlighting the need for dynamic surveillance and adaptive intervention strategies to target evolving dengue hotspots.

**Table 1.** Moran’s I value interpretation.

Moran’s I value	Interpretation
>0.5	Strong positive spatial autocorrelation (high clustering)
0–0.5	Weak positive spatial autocorrelation (some clustering)
−0.5–0	Weak negative spatial autocorrelation (slight dispersion)
<−0.5	Strong negative spatial autocorrelation (high dispersion)

**Table 2.** Moran’s I analysis for dengue (2021–2024).

Year	Moran ‘s I value	p-value	Interpretation
2021	0.243	0.024	Significant positive spatial clustering
2022	0.014	0.323	No significant spatial autocorrelation
2023	0.282	0.007	Significant positive spatial clustering
2024	0.162	0.073	No significant spatial autocorrelation

## LISA significance and cluster analysis of dengue incidence (2021–2024)

**Figure 6.**
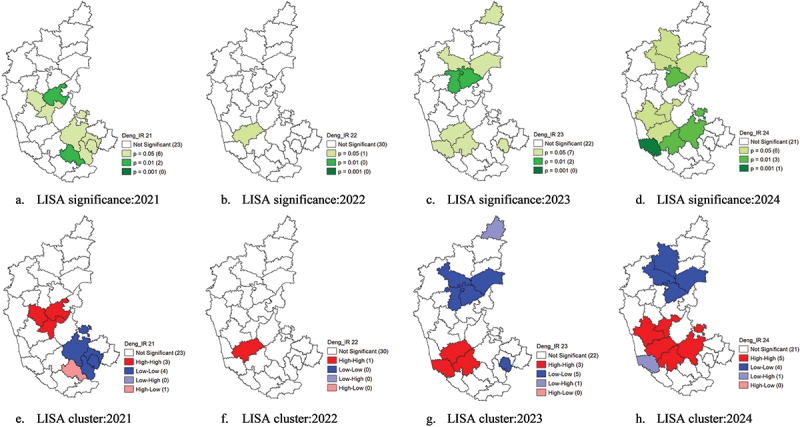
LISA significance (a.d) and LISA cluster maps (e-h) of dengue IR (2021–2024).

The LISA significance and cluster analysis for dengue incidence in Karnataka from 2021 to 2024 were assessed and interpreted as shown in Table 3, which reveals dynamic spatial patterns, with shifts in high-risk and low-risk areas over time (Table 4). In 2021, significant hotspots, i.e. HH clusters, were detected in Davangere (*p* = 0.05), Haveri (*p* = 0.05), and Vijayanagara (*p* = 0.01), indicating localised hotspots of dengue transmission in central Karnataka. Whereas the coldspots, i.e. LL clusters, were identified in Tumakuru (*p* = 0.05), Bengaluru Urban and Bengaluru Rural (*p* = 0.05), and Ramanagara (*p* = 0.05), suggesting consistently lower transmission in these areas. Mandya (*p* = 0.01) emerged as an HL outlier, in Figure 6, indicating a localised outbreak in an otherwise low-risk zone.

**Table 3.** LISA interpretation.

Cluster	Interpretation	Spatial pattern
High-high (HH)/Hotspot	A high-value area surrounded by high values	High-risk area/Concentrated number of cases
Low-Low (LL)/Coldspot	A low-value area surrounded by low values	Low-risk area
High-low (HL)/Outlier	A high-value area surrounded by low values	Localised outbreak
Low-high (LH)/Outlier	A low-value area surrounded by high values	Possible underreporting

**Table 4.** Clusters of dengue incidence.

Year	High-high cluster	Low-low cluster	Low-high cluster	High-low cluster
2021	Davangere, Haveri, Vijayanagara.	Tumkuru, Bengaluru Urban, Bengaluru Rural, Ramanagara.	–	Mandya
2022	Chikkamagaluru	–	–	–
2023	Dakshina Kannada, Hassan, Chikkamagaluru	Bagalkot, Raichur, Koppal, Gadag, Bengaluru Urban	Bidar	–
2024	Davangere, Chikkamagluru, Hassan, Tumkuru, Shivamogga	Vijayapura, Bagalkot, Koppal, Raichur	Dakshina Kannada	–

By 2022, the spatial clustering pattern shifted, with only one HH cluster in Chikkamagaluru (*p* = 0.05), suggesting a localised concentration of dengue cases rather than widespread high-incidence areas. This could indicate effective control measures in previously high-risk districts or a natural decline in cases. However, in 2023, dengue incidence exhibited an expansion of clustering patterns, with HH clusters appearing in Dakshina Kannada (*p* = 0.05), Hassan (*p* = 0.05), and Chikkamagaluru (*p* = 0.05). This marks a southward shift in dengue transmission hotspots, possibly influenced by climatic conditions, urbanisation, or changes in vector breeding habitats. At the same time, LL clusters expanded, with Bagalkot (*p* = 0.05), Raichur (*p* = 0.05), Koppal (*p* = 0.01), Gadag (*p* = 0.01), and Bengaluru Urban (*p* = 0.05) identified as low-risk areas. The stability of these LL clusters suggests sustained control measures or environmental constraints limiting vector proliferation. Bidar (*p* = 0.05) was recognised as an LH outlier, indicating it had lower case counts but was surrounded by high-incidence districts, potentially making it vulnerable to future outbreaks.

In 2024, dengue transmission patterns further evolved, with HH clusters in Davangere (*p* = 0.05), Chikkamagaluru (*p* = 0.05), Hassan (*p* = 0.01), Shivamogga (*p* = 0.05) and Tumakuru (*p* = 0.01). The re-emergence of Davangere as an HH cluster and new HH clusters in Chikkamagaluru and Hassan indicate that central Karnataka remains a key dengue hotspot. The LL clusters persisted in Vijayapura (*p* = 0.05), Bagalkot (*p* = 0.05), Koppal (*p* = 0.01), and Raichur (*p* = 0.05), reinforcing northern Karnataka as a low-risk region, shown in Figure 6. This stability suggests either effective control strategies or environmental conditions unfavourable for mosquito breeding. Dakshina Kannada (*p* = 0.05) was identified as an LH outlier, meaning it had low incidence but was surrounded by high-incidence districts, indicating potential risk for future outbreaks.

These findings highlight the shifting nature of dengue hotspots in Karnataka, with Davangere, Chikkamagaluru, and Hassan emerging as persistent high-risk areas, while northern districts like Vijayapura, Bagalkot, and Raichur remain stable low-risk zones. The southward movement of HH clusters and the identification of LH outliers suggest the need for continued surveillance, early detection systems, and adaptive vector control strategies.

## Spatial autocorrelation of chikungunya IR (2021–2024)

**Figure 7.**
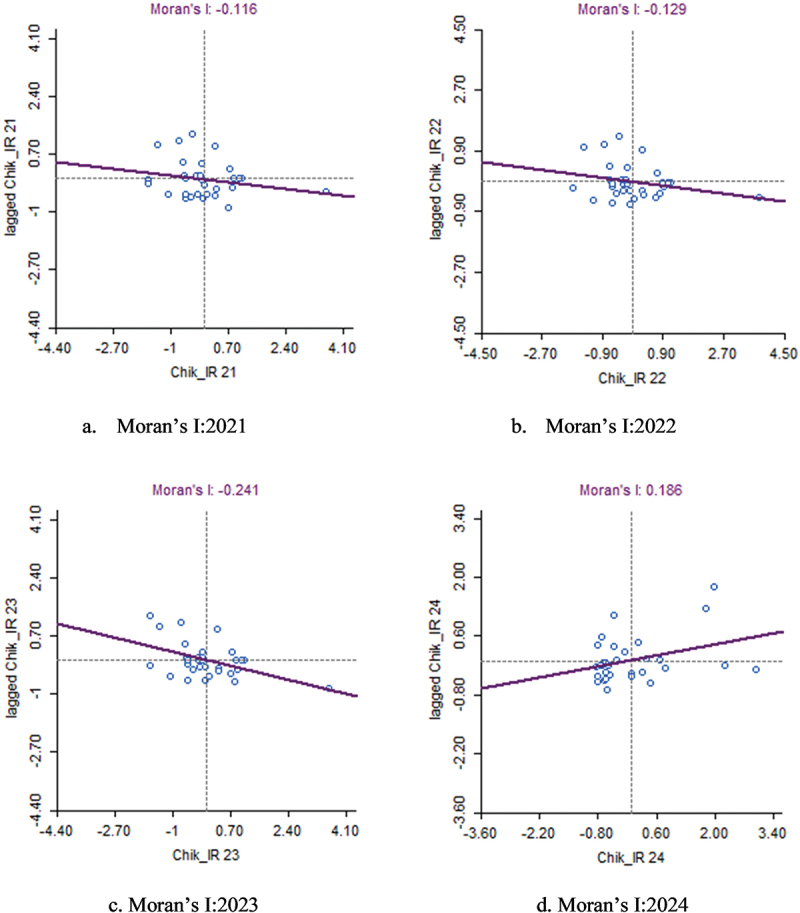
Moran’s I scatter plots (a-d) for chikungunya IR (2021–2024).

The Moran’s I scatter plots for chikungunya IR from 2021 to 2024 indicate shifting spatial autocorrelation trends over the study period. In 2021, Moran’s I value was −0.116, suggesting a weak inverse relationship between chikungunya IR in one district and its neighbouring districts, implying a nearly random spatial distribution. This trend continued in 2022, with a slightly lower Moran’s I value of −0.129, reinforcing the absence of significant clustering or dispersion patterns. In 2023, Moran’s I value declined further to −0.241, indicating a slightly more substantial negative autocorrelation (Table 5). This means districts with high IR were more likely to be surrounded by low IR districts, and vice versa, showing some evidence of dispersed cases rather than clusters. However, by 2024, Moran’s I value shifted to 0.186 indicating a significant positive spatial autocorrelation. This reflects the emergence of spatial clusters, where districts with high or low chikungunya IR tended to be near others with similarly high or low values (Figure 7).

**Table 5.** Moran’s I analysis for chikungunya (2021–2024).

Year	Moran‘s I value	p-value	Interpretation
2021	−0.116	0.205	No significant spatial autocorrelation
2022	−0.129	0.17	No significant spatial autocorrelation
2023	−0.241	0.024	Significant negative spatial autocorrelation
2024	0.186	0.041	Significant positive spatial autocorrelation

These findings suggest that chikungunya incidence in Karnataka did not exhibit consistent spatial dependence across years (interpretation shown in Table 1), with spatial clustering becoming statistically significant only in 2024. The variability in spatial autocorrelation patterns implies that non-spatial factors such as climate variability, vector control measures, and healthcare access may play a more dominant role in influencing chikungunya transmission in most years.

Although the local Moran’s I values suggest weak or no spatial autocorrelation, LISA analysis is still valuable for detecting localised clusters and spatial outliers. Certain districts may exhibit significant high-risk or low-risk clusters even without strong global trends due to local environmental, demographic, or intervention-related factors. Identifying these areas helps target disease control efforts more effectively, ensuring resources are allocated where they are needed most.

## LISA significance and cluster analysis of chikungunya incidence (2021–2024)

Despite the weak local spatial autocorrelation (interpreted referring to Table 3), the LISA significance analysis reveals that certain districts exhibited significant local clustering patterns, indicating localised hotspots and transmission variations over time (Table 6).

**Table 6.** Clusters of chikungunya.

Year	High-high cluster	Low-low cluster	Low-high cluster	High-low cluster
2021	–	Vijayapura	Dakshina Kannada, Mysuru	Yadgir
2022	–	Belgavi, Dharwad	Dakshina Kannada, Mysuru	–
2023	–	Dakshina Kannada, Mysuru	Kodagu	–
2024	Kalburgi	Koppal	Yadgir	–

**Figure 8.**
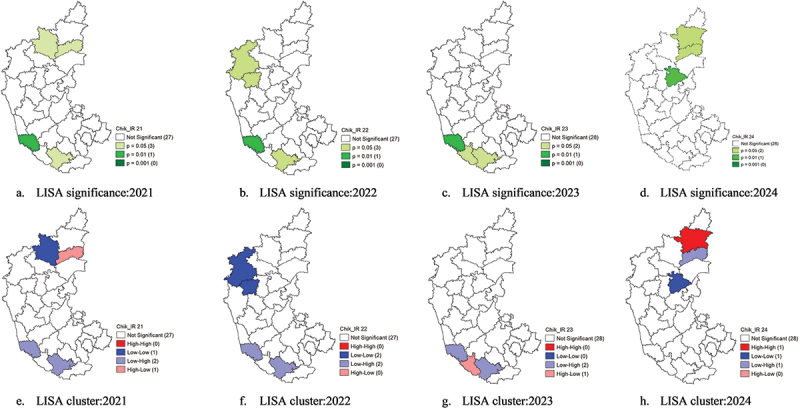
LISA significance (a-d) and LISA cluster maps (e-h) of chikungunya IR (2021–2024).

In 2021, significant clustering was observed in both northern and southern Karnataka. Vijayapura (*p* = 0.05) exhibited an LL cluster (cold spot), indicating consistently low chikungunya incidence surrounded by other low-incidence districts. Dakshina Kannada (*p* = 0.01) and Mysuru (*p* = 0.05) were classified as LH outliers, suggesting that these districts had low incidence but were surrounded by high-incidence neighbours. This pattern highlights potential spillover risks from high-risk districts into low-risk areas. Yadgir (*p* = 0.05) was detected as an HL outlier, indicating a higher chikungunya incidence despite being surrounded by lower-incidence regions, potentially due to localised outbreaks or environmental factors favouring transmission. In 2022, clustering patterns remained relatively stable, but some shifts were observed. The LL clusters expanded to Belagavi (*p* = 0.05) and Dharwad (*p* = 0.05), reinforcing stable low-incidence zones in these districts. Dakshina Kannada (*p* = 0.01) and Mysuru (*p* = 0.05) remained LH outliers, suggesting they continued to be transition zones where cases were spatially linked to neighbouring high-incidence districts, as shown in Figure 8.

By 2023, there was a notable shift in spatial clustering, with a substantial localised increase in cases in certain regions. Dakshina Kannada (*p* = 0.01) and Mysuru (*p* = 0.05) remained LH outliers, indicating that they consistently exhibited low incidence despite neighbouring high-risk districts. Kodagu (*p* = 0.05) emerged as an HL outlier, meaning that a higher-than-expected incidence was recorded despite being surrounded by low-incidence areas. This could be due to environmental or demographic factors, such as increased mosquito breeding sites, human mobility, or changes in vector control measures. By 2024, the spatial clustering pattern changed significantly, with Kalburgi (*p* = 0.05) emerging as an HH cluster, indicating a significant chikungunya hotspot. This suggests that Kalburgi experienced substantially higher incidence rates, with neighbouring districts also showing high case counts, reinforcing a strong spatial autocorrelation in this region. Yadgir (*p* = 0.05) was classified as an LH outlier, meaning that these districts had a lower incidence but were surrounded by high-incidence areas, making them potential risk zones for future outbreaks.

The LISA analysis highlights the shifting nature of chikungunya transmission in Karnataka, with high-incidence hotspots evolving over time. While cold spots in northern districts like Vijayapura, Koppal, Belagavi, and Dharwad suggest stable low-incidence zones, the presence of outliers in Dakshina Kannada, Yadgir, Mysuru, and Kodagu indicates transitional zones where transmission dynamics are changing. The emergence of Kalburgi as a hotspot in 2024 underscores the need for urgent intervention and targeted vector control measures (Figure 8).

## Discussion

The spatial and temporal analysis of dengue and chikungunya incidence in Karnataka from 2021 to 2024 highlights significant trends and evolving disease transmission patterns across the state. The findings indicate an overall increase in dengue cases, with certain districts experiencing disproportionately high IR, while chikungunya displayed lower overall IR with irregular fluctuations and limited spatial clustering in most years. The spatial autocorrelation and LISA cluster analyses reveal dynamic shifts in disease hotspots, emphasising the need for adaptive public health strategies [33,34]. The divergent spatial autocorrelation patterns observed for dengue (moderate positive clustering) and chikungunya (weak or negative autocorrelation) may be attributed to differences in vector dynamics, human mobility, and immunity profiles. Dengue often exhibits pronounced clustering due to repeated outbreaks in the same regions, driven by established *Aedes* populations and local transmission chains [35]. In contrast, chikungunya may display more sporadic spatial distribution due to lower baseline endemicity, episodic introductions, and rapid outbreak containment [17]. These differences may also reflect variation in surveillance sensitivity or diagnostic focus across districts. The contrast with the Punjab study (2012–2019), which reported clustering for chikungunya, could stem from regional variations in vector ecology, population density, or climatic conditions [36]. Similarly, the lack of clustering reported in the Barbados study may relate to uniform transmission risk across a smaller geographic area or differences in health system response [37]. These comparisons underscore the need for locally adaptive public health strategies, as spatial patterns of arboviral diseases are shaped by complex and region-specific interactions between environment, host, and vector.

The year-wise trend of dengue and chikungunya cases in Karnataka reveals distinct yet interconnected disease transmission patterns. Dengue cases surged sharply in 2024, nearly doubling from previous years, suggesting an interplay of changing environmental conditions, increased vector breeding, and improved surveillance. The IR analysis highlights spatial heterogeneity, with districts such as Mandya, Shivamogga, Chikkamagaluru, Udupi and Mysuru consistently reporting high IR, likely due to favourable ecological conditions, including urbanisation, stagnant water sources, and climatic factors like temperature and humidity that support *Aedes* mosquito proliferation [38]. Conversely, despite its high population density, Bengaluru Urban recorded the lowest IR values, possibly due to better healthcare infrastructure and vector control measures. However, its increasing trend from 2021 to 2024 indicates a gradual rise in dengue transmission, necessitating continued monitoring.

Similarly, chikungunya incidence exhibited irregular fluctuations over the study period, with localised fluctuations, while some districts, such as Mandya and Kolara, saw a decline in cases, whereas Shivamogga exhibited persistent transmission. Emerging cases in Raichur suggest the potential for outbreaks in previously low-risk areas, emphasising the importance of continuous surveillance. Unlike dengue, which displayed a notable geographic expansion, chikungunya transmission appeared more localised, with endemic foci maintaining steady IR.

A broader spatial analysis indicates that the dengue burden remains higher in southern Karnataka, while certain northern districts, such as Kalaburagi, Koppal, Dharwad, Haveri and Vijayapura, are increasing. In contrast, chikungunya follows a more stable yet regionally concentrated pattern. These findings highlight the need for targeted vector control and surveillance strategies tailored to the transmission dynamics of both diseases, ensuring early detection and effective intervention across diverse ecological settings. Many endemic areas shared borders with Karnataka, i.e. Maharashtra, Goa, Kerala, Tamil Nadu, Andhra Pradesh and Telangana, which are also dengue prone. The study suggested that neighbouring regions with similar ecology, human behaviour, and social patterns could influence dengue transmission, leading to spatial associations [39].

The spatial autocorrelation analysis of dengue incidence from 2021 to 2024 revealed moderate positive clustering, except in 2022 and 2024, where cases were randomly distributed, possibly due to widespread outbreaks affecting both high- and low-risk districts equally. LISA cluster analysis identified shifting dengue hotspots, with Davangere, Haveri, and Vijayanagara in 2021 and Dakshina Kannada, Hassan, and Chikkamagaluru in 2023. By 2024, new high-risk clusters emerged in central Karnataka, while northern districts remained low-risk, likely due to effective vector control or unfavourable mosquito breeding conditions.

Unlike dengue, chikungunya displayed weak spatial autocorrelation, with cases randomly distributed across Karnataka. However, localised hotspots emerged, such as Kodagu in 2023 and Yadgir as an LH outlier in 2024, suggesting potential spillover risks. Vijayapura, Koppal, Belagavi, Dharwad, Mysuru and Dakshina Kannada were identified as unstable cold spots, indicating low transmission for different years in the state. The spatial randomness of chikungunya suggests outbreaks may be driven by localised factors rather than large-scale regional transmission [13].

The increasing dengue burden in high-risk districts underscores the need for sustained vector control and GIS-based surveillance for real-time hotspot detection [4,40,41]. Integrating climatic data, socio-demographic and land-use mapping can enhance outbreak prediction and response strategies [42,43]. The integration of artificial intelligence (AI) and machine learning (ML) with GIS can enhance disease surveillance by enabling predictive modelling, automated hotspot detection, and dynamic risk mapping. These tools can analyse complex spatio-temporal patterns using real-time data, improving the precision and responsiveness of public health interventions [44,45].

The COVID-19 pandemic likely influenced dengue and chikungunya case reporting due to disrupted surveillance, resource diversion, and reduced healthcare-seeking behaviour. This may have led to underreporting or delays in detection, particularly in 2020–2022, affecting the interpretation of trends during the study period [9]. Potential misclassification and underreporting, due to the similarity in symptoms of dengue and chikungunya, may influence spatial distribution patterns [14,46]. Some cases may go unreported as mild symptoms are often ignored, highlighting the need for improved diagnosis and reporting to refine spatial analysis and public health interventions [47–49].

Spatial analysis using GIS provided critical insights into the geographic distribution of dengue and chikungunya, identifying high-risk districts and transmission clusters [40]. While dengue exhibited spatial clustering, chikungunya cases were more randomly distributed, indicating distinct transmission dynamics. Moran’s I and LISA cluster analysis helped pinpoint persistent hotspots, guiding targeted vector control and surveillance [33,50]. However, potential underreporting and symptom overlap may affect spatial patterns, emphasising the need for accurate disease detection. GIS-based mapping is an essential tool in disease surveillance, allowing for real-time monitoring, resource optimisation, and predictive modelling [51]. This approach is valuable for vector-borne diseases and broader public health applications, enabling data-driven decision-making and effective outbreak management [52–55]. This study contributes to the global agenda on vector-borne disease control by demonstrating the applicability of GIS-based spatial analysis in identifying high-risk areas and guiding targeted interventions. The findings emphasise the need for policy-driven implementation of real-time spatial surveillance, which can enhance outbreak preparedness, resource allocation, and vector management strategies at regional and global levels.

## Conclusion

The spatial analysis of dengue and chikungunya cases in Karnataka reveals distinct geographic clustering, reinforcing the need for spatially targeted interventions rather than uniform control strategies. The use of spatial autocorrelation and cluster analysis in this study identifies persistent hotspots, enabling district health departments and municipal authorities to prioritise high-risk zones for focused interventions such as intensified fogging, source reduction, community education, and to anticipate outbreak risks. This GIS-based approach has practical, real-time applications. District health departments can integrate spatial analysis with digital surveillance platforms to develop automated early warning systems, enabling rapid response to emerging clusters. The methods demonstrated in this study are scalable and adaptable, offering valuable input for both national and global frameworks on integrated disease surveillance and vector control. By identifying high-risk areas and spatial patterns of transmission, this study provides actionable insights for policymakers and other relevant stakeholders, including local authorities, to design more effective, evidence-based, and locally tailored interventions to combat transmission of dengue and chikungunya. Strengthening interdisciplinary collaboration between epidemiologists, climatologists, entomologists, remote sensing and GIS experts, data scientists, and urban planners, alongside community engagement, will be essential for building adaptive, predictive, and data-driven systems.

## Limitations

While this study offers valuable insights into the spatial clustering of dengue and chikungunya in Karnataka, several limitations are acknowledged:

- **Use of secondary data**: The analysis relies on secondary data from government health surveillance systems, which may be subject to underreporting, particularly in rural or under-resourced regions with limited access to diagnostic infrastructure.
- **Potential reporting bias and case misclassification**: Since no specific treatment exists for dengue or chikungunya, many cases are managed symptomatically without laboratory confirmation. This can lead to reporting bias and potential misclassification, especially due to the clinical similarity between the two diseases. As a result, the spatial accuracy of case mapping may be affected.
- **Limitations of spatial autocorrelation methods**: Techniques like Moran’s I and LISA effectively identify clustering and hotspots but do not imply causation or account for potential confounding variables.

## Future scope

Future studies may incorporate additional factors such as land use/land cover, population density, water storage patterns, and socioeconomic factors to better understand the ecological and structural determinants of disease transmission. Such multi-dimensional analysis can enhance the explanatory power of spatial models and inform more equitable public health interventions. Coupling GIS with AI and ML can further enhance outbreak forecasting by analysing historical trends, climate variability, and population movement patterns for any disease surveillance.

## Supplementary Material

Checklist.docx

## Data Availability

The data used in this study were obtained from publicly available government health records and surveillance reports. All other data generated or analysed during this study are included in this published article.

## Author confirmation

Permission was obtained from all individuals mentioned in the acknowledgements section to include their names and affiliations in this manuscript.

## References

[1] Sharma P, Pavitra K, Shettigar S. Trends in dengue virus infection with seasonal variation at a tertiary care centre, Mangaluru: a retrospective study. IP Intl J Med Microbiol Trop Dis. 2022;8:294–17. doi: 10.18231/j.ijmmtd.2022.057

[2] Shankar P, Mahamud S. Clinical profile and laboratory characteristics of dengue fever in children: analysis of 2019 outbreak from Bengaluru, Karnataka, India. Int J Contemp Pediatr. 2020;7:1670–1676. doi: 10.18203/2349-3291.ijcp20203160

[3] Barbosa RMR, de melo-Santos MAV, Silveira JC Jr, et al. Infestation of an endemic arbovirus area by sympatric populations of Aedes aegypti and Aedes albopictus in Brazil. Mem Inst Oswaldo Cruz. 2020;115:e190437. doi: 10.1590/0074-02760190437 32428083 PMC7233267

[4] H C V, Sn N, M K J, et al. Unraveling dengue dynamics: in-depth epidemiological and entomological analyses in Bengaluru, India. J Trop Med. 2024;2024:1–7. doi: 10.1155/2024/7247263 PMC10874290 38371747

[5] National Centre for Vector Borne Diseases Control (NCVBDC). Dengue cases and deaths in India [Internet]. New Delhi: Ministry of Health and Family Welfare, Government of India; [cited 2024 Nov 2]. Available from: https://ncvbdc.mohfw.gov.in/index1.php?lang=1&level=1&sublinkid=5776&lid=3690

[6] Gubler DJ. Dengue and dengue hemorrhagic fever. Clin Microbiol Rev. 1998;11:480–496. doi: 10.1128/CMR.11.3.480 9665979 PMC88892

[7] Intergovernmental Panel on Climate Change (IPCC). Climate change 2023: synthesis report. Geneva: IPCC; 2023 [cited 2025 Jul 3]. Available from: https://www.ipcc.ch/report/ar6/syr/

[8] Dharmamuthuraja D, Rohini PD, Iswarya Lakshmi M, et al. Determinants of Aedes mosquito larval ecology in a heterogeneous urban environment: a longitudinal study in Bengaluru, India. PLOS Negl Trop Dis. 2023;17:e0011704. doi: 10.1371/journal.pntd.0011702 PMC10659209 37939204

[9] Reegan DA, Gandhi MR, Asharaja AC, et al. COVID-19 lockdown: impact assessment on Aedes larval indices, breeding habitats, effects on vector control programme and prevention of dengue outbreaks. Heliyon. 2020;6:e05181. doi: 10.1016/j.heliyon.2020.e05181 33043162 PMC7534600

[10] Mutheneni SR, Morse AP, Caminade C, et al. Dengue burden in India: recent trends and importance of climatic parameters. Emerg Microbes Infect. 2017;6:1–10. doi: 10.1038/emi.2017.57 PMC5583666 28790459

[11] Mani S, Ghosh S, Sharma R, et al. Controlling dengue, an urban pandemic: a case study of Delhi, India. In: Katz R, Boyce M, editors. Inoculating cities: case studies of urban pandemic preparedness. Cambridge (MA): Elsevier; 2021. p. 1–19. doi: 10.1016/B978-0-12-820204-3.00001-2

[12] Costa LB, Barreto FKA, Barreto MCA, et al. Epidemiology and economic burden of chikungunya: a systematic literature review. Trop Med Infect Dis. 2023;8:301. doi: 10.3390/tropicalmed8060301 37368719 PMC10302198

[13] Huber JH, Childs ML, Caldwell JM, et al. Seasonal temperature variation influences climate suitability for dengue, chikungunya, and Zika transmission. PLOS Negl Trop Dis. 2018;12:e0006451. doi: 10.1371/journal.pntd.0006451 29746468 PMC5963813

[14] De Almeida PMP, Nobre AA, Câmara DCP, et al. Dengue, chikungunya, and Zika: spatial and temporal distribution in Rio de Janeiro state, 2015–2019. Trop Med Infect Dis. 2022;7:141. doi: 10.3390/tropicalmed7070141 35878153 PMC9318038

[15] Dzul-Manzanilla F, Correa-Morales F, Che-Mendoza A, et al. Identifying urban hotspots of dengue, chikungunya, and Zika transmission in Mexico to support risk stratification efforts: a spatial analysis. Lancet Planet Health. 2021;5:e277–e285. doi: 10.1016/S2542-5196(21)00030-9 33964237 PMC8114339

[16] Ganesan VK, Duan B, Reid SP. Chikungunya virus: pathophysiology, mechanism, and modeling. Viruses. 2017;9:368. doi: 10.3390/v9120368 29194359 PMC5744143

[17] Translational research consortia (TRC) for chikungunya virus in India. Current status of chikungunya in India. Front Microbiol. 2021;12:69s5173. doi: 10.3389/fmicb.2021.695173 PMC8274422 34262552

[18] Queiroz ERDS, Medronho RA, Giovanetti M. Overlap between dengue, Zika and chikungunya hotspots in the city of Rio de Janeiro. PLOS ONE. 2022;17:e0273980. doi: 10.1371/journal.pone.0273980 36067192 PMC9447914

[19] Palaniyandi M. The environmental aspects of dengue and chikungunya outbreaks in India: gIS for epidemic control. Int J Mosq Res. 2014;1:35–40.

[20] Horwood PF, Buchy P. Chikungunya. Rev Sci Tech. 2015;34:479–489. doi: 10.20506/rst.34.2.2373 26601450

[21] Butt MA, Khalid A, Ali A, et al. Towards a web GIS-based approach for mapping a dengue outbreak. Appl Geomat. 2020;12:121–131. doi: 10.1007/s12518-019-00282-7

[22] Ashwini M, Talluri Rameshwari KR, Sumana MN, et al. Gis-based analysis of the spatial distribution of dengue disease in Mysuru district and India, 2013–2018. Int J Mosq Res. 2020;7:13–26. doi: 10.22271/23487941.2020.v7.i6a.484

[23] Tang MYF, Tsoi CW. Gis initiatives in improving the dengue vector control. In: Lai P Mak A, editors. Gis for health and the environment. Berlin, Heidelberg: Springer; 2007. p. 211–226. doi: 10.1007/978-3-540-71318-0_13

[24] Balaji D, Saravanabavan V. Geospatial variation of dengue risk zone in Madurai city using autocorrelation techniques. GeoJournal. 2021;86:1481–1501. doi: 10.1007/s10708-020-10143-1

[25] Romero Canal M, da Silva Ferreira ER, Estofolete CF, et al. Spatiotemporal-based clusters as a method for dengue surveillance. Rev Panam Salud Publica. 2017;41:1–6. doi: 10.26633/RPSP.2017.162 PMC6645192 31384275

[26] McLafferty S. Disease cluster detection methods: recent developments and public health implications. Ann Gis. 2015;21:127–133. doi: 10.1080/19475683.2015.1008572

[27] Le TT, Nguyen HT, Vu PT, et al. Space-time scanning statistics in the prediction and evaluation of dengue epidemic clusters. IJID Regions. 2024;13:100441. doi: 10.1016/j.ijregi.2024.100441 39351397 PMC11440294

[28] Uwishema O. Addressing the effects of the earthquakes on Türkiye’s health-care system. Lancet. 2023;401:727. doi: 10.1016/S0140-6736(23)00326-4 10378 38888885

[29] Pandya K, Bhatti VK, Ghosh S, et al. Community-based study to describe the epidemiology of dengue infection in a large cantonment during one transmission season. Med J Armed Forces India. 2024;80:276–280. doi: 10.1016/j.mjafi.2023.04.008 38799995 PMC11116983

[30] Department of health & family Welfare, Government of Karnataka. Dengue & chikungunya – malaria & H1N1 reports [internet]. Bengaluru: Government of Karnataka; [cited 2025 Jan 13]. Available from: https://hfwcom.karnataka.gov.in/info-4/Dengue+&+Chikungunya±±Malaria+&+H1N1-Reports/en

[31] Government of Karnataka. Area and population – general statistics [internet]. Bengaluru: Department of State Excise, Government of Karnataka; [cited 2025 Jul 3]. Available from: https://stateexcise.karnataka.gov.in/info-4/General+Statistics/Area+and+population/en

[32] Karnataka State Remote Sensing Applications Centre. Karnataka Geographic Information System (K-GIS) [internet]. Bengaluru: K-GIS; [cited 2024 Nov 12]. Available from: https://kgis.ksrsac.in

[33] Zhu B, Fu Y, Liu J, et al. Detecting the priority areas for health workforce allocation with LISA functions: an empirical analysis for China. BMC Health Serv Res. 2018;18:957. doi: 10.1186/s12913-018-3737-y 30541543 PMC6292090

[34] Hu W, Clements A, Williams G, et al. Spatial analysis of notified dengue fever infections. Epidemiol Infect. 2011;139:391–399. doi: 10.1017/S0950268810000713 20392302

[35] Verma P, Baskey U, Choudhury KR, et al. Changing pattern of circulating dengue serotypes in the endemic region: an alarming risk to the healthcare system during the pandemic. J Infect Public Health. 2023;16:2046–2057. doi: 10.1016/j.jiph.2023.10.014 37944366

[36] Verma M, Panwar S, Sahoo SS, et al. Mapping the stability of febrile illness hotspots in Punjab from 2012 to 2019: a spatial clustering and regression analysis. BMC Public Health. 2023;23:2014. doi: 10.1186/s12889-023-16930-y 37845663 PMC10580620

[37] Lippi CA, Stewart-Ibarra AM, Romero M, et al. Spatiotemporal tools for emerging and endemic disease hotspots in small areas: an analysis of dengue and chikungunya in Barbados, 2013–2016. Am J Trop Med Hyg. 2020;103:149–156. doi: 10.4269/ajtmh.19-0919 32342853 PMC7356414

[38] Kamath R, Gupta R, Chandrasekaran V, et al. Assessment of environmental factors associated with dengue transmission in Udupi Taluk, Karnataka. J Sci Soc. 2013;40:159. doi: 10.4103/0974-5009.120060

[39] Santana LMR, Baquero OS, Maeda AY, et al. Spatio-temporal dynamics of dengue-related deaths and associated factors. Rev Inst Med Trop Sao Paulo. 2022;64:e30. doi: 10.1590/S1678-9946202264030 35384961 PMC8993154

[40] Martínez-Bello D, López-Quílez A, Prieto AT. Spatiotemporal modeling of relative risk of dengue disease in Colombia. Stoch Environ Res Risk Assess. 2018;32:1587–1601. doi: 10.1007/s00477-017-1461-5

[41] Ha TA, León TM, Lalangui K, et al. Household-level risk factors for Aedes aegypti pupal density in Guayaquil, Ecuador [preprint]. bioRxiv. 2020. doi: 10.1101/2020.11.23.391938 PMC8425057 34493321

[42] Siljander M, Uusitalo R, Pellikka P, et al. Spatiotemporal clustering patterns and sociodemographic determinants of COVID-19 (SARS-CoV-2) infections in Helsinki, Finland. Spat Spatiotemporal Epidemiol. 2022;41:100493. doi: 10.1016/j.sste.2022.100493 35691637 PMC8817446

[43] Ganguly KS, Chattopadhyay AK, Dutta A, et al. Spatial clustering of dengue fever: a baseline study in the city of Kolkata. Int J Healthc Med Sci. 2018;4:1–8.

[44] Anno S, Hara T, Kai H, et al. Spatiotemporal dengue fever hotspots associated with climatic factors in Taiwan, including outbreak predictions based on machine-learning. Geospat Health. 2019;14:183–194. doi: 10.4081/gh.2019.771 31724367

[45] Attaway DF, Jacobsen KH, Falconer A, et al. Risk analysis for dengue suitability in Africa using the ArcGIS predictive analysis tools (PA tools). Acta Trop. 2016;158:248–257. doi: 10.1016/j.actatropica.2016.02.018 26945482

[46] Barreto FK, Alencar CH, Araújo FM, et al. Seroprevalence, spatial dispersion and factors associated with flavivirus and chikungunya infection in a risk area: a population-based seroprevalence study in Brazil. BMC Infect Dis. 2020;20:881. doi: 10.1186/s12879-020-05611-5 33234110 PMC7685300

[47] Usman M, Reddy US, Siddiqui LA, et al. Exploration of spatial clustering in maternal health continuum of care across districts of India: a geospatial analysis of demographic and health survey data. PLOS ONE. 2022;17:e0279117. doi: 10.1371/journal.pone.0279117 36520872 PMC9754170

[48] Rosvita NA, Kania N, Suhartono E, et al. Spatial-temporal distribution of dengue in Banjarmasin, Indonesia from 2016 to 2020. Int J Public Health Sci. 2022;11:1167–1175. doi: 10.11591/ijphs.v11i4.21780

[49] Valson JS, Soman B. Ijphs. spatiotemporal clustering of dengue cases in Thiruvananthapuram district, Kerala. Indian J Public Health. 2017;61:74–80. doi: 10.4103/ijph.IJPH_26_16 28721955

[50] Zhu B, Fu Y, Liu J, et al. Spatial distribution of 12 class B notifiable infectious diseases in China: a retrospective study. PLOS ONE. 2018;13:e0195568. doi: 10.1371/journal.pone.0195568 29621351 PMC5886686

[51] Yin S, Ren C, Shi Y, et al. A systematic review on modeling methods and influential factors for mapping dengue-related risk in urban settings. Int J Environ Res Public Health. 2022;19:15265. doi: 10.3390/ijerph192215265 36429980 PMC9690886

[52] Dong B, Khan L, Smith M, et al. Spatio-temporal dynamics of three diseases caused by Aedes-borne arboviruses in Mexico. Commun Med (lond). 2022;2:134. doi: 10.1038/s43856-022-00192-7 36317054 PMC9616936

[53] Talukdar R, Kanungo S, Kitahara K, et al. Identifying clustering of cholera cases using geospatial analysis in Kolkata and surrounding districts: data from patients at tertiary care referral hospitals. Lancet Reg Health Southeast Asia. 2024;31:100510. doi: 10.1016/j.lansea.2024.100510 39640000 PMC11617701

[54] Pandey BW, Yadav G, Tripathi N, et al. Reproductive and child health transition among selected empowered action groups states of India: a district-level analysis. PLOS ONE. 2024;19:e0301587. doi: 10.1371/journal.pone.0301587 38857210 PMC11164384

[55] Wu Y, Wang T, Zhao M, et al. Spatiotemporal cluster patterns of hand, foot, and mouth disease at the province level in mainland China, 2011–2018. PLOS ONE. 2022;17:e0270061. doi: 10.1371/journal.pone.0270061 35994464 PMC9394824

