# Supplementary material for: Spatial epidemiology of dengue and chikungunya in Karnataka using GIS-based analysis
Source: Glob Health Action. 2025 Aug 21;18(1):2543198. doi: 10.1080/16549716.2025.2543198 (PMC12372493; doi:10.1080/16549716.2025.2543198)
Supplement: Checklist.docx [file ZGHA_A_2543198_SM8531.docx]

STROBE Statement—checklist of items that should be included in reports of observational studies

|  | Item No. | Recommendation | Page  No. | Relevant text from manuscript |
| --- | --- | --- | --- | --- |
| **Title and abstract** | 1 | (a) Indicate the study’s design with a commonly used term in the title or the abstract | 1 | Included in the abstract:  **“**This GIS-based cross-sectional study analysed” … |
|  |  | (b) Provide in the abstract an informative and balanced summary of what was done and what was found | 1-2 | Included in the abstract: background, methods, results, and conclusion |
| Introduction | | | |  |
| Background/rationale | 2 | Explain the scientific background and rationale for the investigation being reported | 2-4 | Included in the Introduction section: Explains the burden of vector-borne diseases and the need for spatial analysis. |
| Objectives | 3 | State specific objectives, including any prespecified hypotheses | 1 & 4 | The objective is clearly stated in the abstract and introduction. |
| Methods | | | |  |
| Study design | 4 | Present key elements of study design early in the paper | 4 | Described in the Methods section:Study design: “This study employed a **cross-sectional design** using **GIS-based spatial analysis”….** |
| Setting | 5 | Describe the setting, locations, and relevant dates, including periods of recruitment, exposure, follow-up, and data collection | 4-6 | Included: Study area and data collection: Karnataka state; data from 2021–2024; locations and periods mentioned. |
| Participants | 6 | (a) Cohort study—Give the eligibility criteria, and the sources and methods of selection of participants. Describe methods of follow-up  Case-control study—Give the eligibility criteria, and the sources and methods of case ascertainment and control selection. Give the rationale for the choice of cases and controls  Cross-sectional study—Give the eligibility criteria and the sources and methods of selection of participants | 5 | Cross-sectional. Included in data collection: All reported cases from government health surveillance included (secondary data) |
|  |  | (b) Cohort study—For matched studies, give matching criteria and number of exposed and unexposed  Case-control study—For matched studies, give matching criteria and the number of controls per case | - | - |
| Variables | 7 | Clearly define all outcomes, exposures, predictors, potential confounders, and effect modifiers. Give diagnostic criteria, if applicable | 5-6 | Dengue and chikungunya incidence rates; spatial indicators like Moran’s I. |
| Data sources/ measurement | 8* | For each variable of interest, give sources of data and details of methods of assessment (measurement). Describe comparability of assessment methods if there is more than one group | 5-6 | Government surveillance data: GIS and spatial statistical techniques described |
| Bias | 9 | Describe any efforts to address potential sources of bias | 21 | Secondary data limitations are discussed in the limitations section. |
| Study size | 10 | Explain how the study size was arrived at | 5 | Data were not sampled; complete district-level surveillance data were publicly available only from **2021 to 2024**. |

| Quantitative variables | 11 | Explain how quantitative variables were handled in the analyses. If applicable, describe which groupings were chosen and why | 5 | Incidence Rate per 1,000 population used, grouped by district and year |
| --- | --- | --- | --- | --- |
| Statistical methods | 12 | (a) Describe all statistical methods, including those used to control for confounding | 5-6 | Moran’s I, cluster analysis, and GIS mapping are detailed; no sampling strategy or sensitivity analysis is needed. |
|  |  | (b) Describe any methods used to examine subgroups and interactions | 5-6 |  |
|  |  | (c) Explain how missing data were addressed | - |  |
|  |  | (d) Cohort study—If applicable, explain how loss to follow-up was addressed  Case-control study—If applicable, explain how matching of cases and controls was addressed  Cross-sectional study—If applicable, describe analytical methods taking account of sampling strategy | - |  |
|  |  | (e) Describe any sensitivity analyses | - |  |
| Results | | | | |
| Participants | 13* | (a) Report numbers of individuals at each stage of study—eg numbers potentially eligible, examined for eligibility, confirmed eligible, included in the study, completing follow-up, and analysed | 7 | Descriptive data was reported; the flow diagram does not apply to secondary data. |
|  |  | (b) Give reasons for non-participation at each stage | - |  |
|  |  | (c) Consider use of a flow diagram | - |  |
| Descriptive data | 14* | (a) Give characteristics of study participants (eg demographic, clinical, social) and information on exposures and potential confounders | 7 | District-level incidence data reported; missing data not applicable |
|  |  | (b) Indicate number of participants with missing data for each variable of interest | - |  |
|  |  | (c) Cohort study—Summarise follow-up time (eg, average and total amount) | - |  |
| Outcome data | 15* | Cohort study—Report numbers of outcome events or summary measures over time | - |  |
|  |  | Case-control study—Report numbers in each exposure category, or summary measures of exposure | - |  |
|  |  | Cross-sectional study—Report numbers of outcome events or summary measures | 5-7 | Incidence rates and counts presented |
| Main results | 16 | (a) Give unadjusted estimates and, if applicable, confounder-adjusted estimates and their precision (eg, 95% confidence interval). Make clear which confounders were adjusted for and why they were included | 12-19 | Spatial autocorrelation and cluster analysis results included |
|  |  | (b) Report category boundaries when continuous variables were categorized | - |  |
|  |  | (c) If relevant, consider translating estimates of relative risk into absolute risk for a meaningful time period | - |  |

| Other analyses | 17 | Report other analyses done—eg analyses of subgroups and interactions, and sensitivity analyses | 12-19 | Cluster patterns and spatial maps interpreted. |
| --- | --- | --- | --- | --- |
| Discussion | | | | |
| Key results | 18 | Summarise key results with reference to study objectives | 19 | Included: The discussion includes summary of the findings |
| Limitations | 19 | Discuss limitations of the study, taking into account sources of potential bias or imprecision. Discuss both direction and magnitude of any potential bias | 21 | Bias from secondary data and lack of individual-level variables discussed are mentioned under limitations. |
| Interpretation | 20 | Give a cautious overall interpretation of results considering objectives, limitations, multiplicity of analyses, results from similar studies, and other relevant evidence | 19-21 | Results contextualised with public health relevance and other studies, mentioned in discussion section |
| Generalisability | 21 | Discuss the generalisability (external validity) of the study results | 21 | Applicable to similar vector-borne disease/other infection settings globally, mentioned in conclusions. |
| Other information | |  | | |
| Funding | 22 | Give the source of funding and the role of the funders for the present study and, if applicable, for the original study on which the present article is based | 22 | No funding was received; the funding section declares this. |

*Give information separately for cases and controls in case-control studies and, if applicable, for exposed and unexposed groups in cohort and cross-sectional studies.

**Note:** An Explanation and Elaboration article discusses each checklist item and gives methodological background and published examples of transparent reporting. The STROBE checklist is best used in conjunction with this article (freely available on the Web sites of PLoS Medicine at http://www.plosmedicine.org/, Annals of Internal Medicine at http://www.annals.org/, and Epidemiology at http://www.epidem.com/). Information on the STROBE Initiative is available at www.strobe-statement.org.
